# Taking a Climate and Health History: A One Health-Informed Approach to Primary Care and Services

**DOI:** 10.5334/aogh.4898

**Published:** 2026-01-08

**Authors:** Firoz Abdoel Wahid, Maureen Lichtveld, Samantha Totoni, Brian Earle

**Affiliations:** 1 University of Pittsburgh School of Public Health, 130 DeSoto Street, Pittsburgh, PA, USA

**Keywords:** climate and health history, one health, primary care services, community health workers

## Abstract

Climate change poses the greatest public health threat, disproportionately impacting communities in Low- and Middle-Income Countries (LMICs) where fragile health systems increase vulnerability. Despite this, clinical practice often overlooks climate-related health risks. Current approaches focus on single disciplines or settings, limiting broader integration. Incorporating a One Health approach—recognizing the interconnection of human, animal, environmental, and plant health—into routine clinical encounters offers a pathway to strengthen climate-health awareness. This manuscript presents practical guidance for integrating climate and health histories, with a focus on heat exposure, and emphasizes the role of physicians, other health providers and three categories of Community Health Workers (CHWs) across the care continuum. A case study illustrates how targeted climate and environmental inquiries during history-taking can advance diagnosis and patient education. Embedding One Health in clinical care bridges existing gaps, enhances early detection of climate-related illness, and promotes culturally sensitive, holistic health interventions in vulnerable communities.

## Background

Climate change is widely recognized as a fundamental threat to public health [1]. Addressing this challenge requires an urgent and holistic approach from healthcare providers and other health professionals. The Intergovernmental Panel on Climate Change (IPCC) Sixth Assessment Report (AR6), as well as the Lancet Countdown on Health and Climate Change, identified numerous health impacts associated with climate change, including heat stress, cardiovascular and respiratory distress, vector-borne diseases, zoonoses, waterborne and water-related diseases, malnutrition, and mental health challenges related to stress and trauma [2, 3]. These impacts can be grouped into four overarching themes posing the most urgent threats: infectious disease, extreme heat, food and water safety and security, and poor air quality [4]. Each threat may be associated with mental health challenges. Populations at disproportionate risk include older adults, infants and children, pregnant individuals, people with chronic diseases, outdoor workers, people with disabilities, Indigenous and Tribal Peoples, and those living in poverty or informal settlements. Recognizing these inequities is essential for guiding adaptation and mitigation strategies in clinical and community settings [3]. Climate change affects people differently across the lifespan. Climate change creates distinct risks across the lifespan—from prenatal vulnerability to heat and pollution, through childhood susceptibility due to immature physiology, to adolescent mental health and heat-stress risks. Working-age adults often face occupational exposures, while older adults are more affected by chronic illness and limited mobility [5]. While recognizing these age-specific vulnerabilities is essential, this article will focus on adults.

One Health is a collaborative, transdisciplinary approach recognizing that the health of people is inextricably connected to that of animals, plants, and our shared environment [6]. In the clinical setting, One Health principles offer a practical framework for incorporating environmental and animal exposure considerations into patient care. Integrating a One Health approach into climate and health history-taking encourages attention to environmental exposures, interactions with pets and other animals, vector contact, and plant-related risks.

Although guidance for climate-related history-taking exists across various specialties and settings, it is often fragmented. In one study, only 26% of participating healthcare providers routinely screened for climate change-related health risks, and just 20% discussed the risks in clinical encounters [7]. The current literature underscores the relevance of climate-related history-taking across a broad spectrum of health professionals, including physician assistants, nurses, midwives, and emergency room practitioners [8–12]. However, while promising progress has been made in addressing factors such as heat stress in clinical settings in High-Income Countries (HIC), health professionals practicing in Low- and Middle-Income Countries (LMICs), lag behind. Although progress is underway in developing e-learning modules, short courses, and integrating climate and health topics into health professions curricula, practical applications for clinical encounters are still limited, especially outside HICs [10–14].

In LMICs, a practical way to integrate climate and health history-taking in primary care is by embedding community health workers (CHWs) on the frontline. Covert et al. [13] developed a framework that categorizes CHWs based on training, work setting, and scope of practice. This framework addresses the significant variability in CHWs’ scope of work, nomenclature, and training. CHWs, in the context of climate change, can work at three levels: promoting a continuum of engagement from prevention and education (Category 1) to intervention and access to care (Category 2), to disease-specific management (Category 3).

The purpose of this viewpoint is to provide a practical, climate-informed framework (especially geared toward extreme heat) for frontline providers and community-based practitioners, including CHWs—particularly those in LMICs—grounded in a One Health approach.

## Areas of Assessment—Climate and Health History-Taking

A climate and health history seeks to ascertain a patient’s health risks, exposures, and vulnerabilities, to arrive at comprehensive clinical management. Key areas to assess include:

- **Environmental Exposure:** Where does the patient live and work? What are typical daily temperatures? Is there access to cooling (e.g., shade, fans, air conditioning)?
- **Occupation:** Does their work involve heat-exposed outdoor (e.g., agriculture, construction) labor, or indoor (e.g., manufacturing plants, commercial kitchens) labor?
- **Housing Conditions:** Is their home constructed to withstand extreme heat or flooding? What materials are used?
- **Animal and Vector Exposure:** Are there animals nearby (e.g., livestock, domestic pets) that may also experience heat-related stress or contribute to zoonotic risks?
- **Social Determinants:** Does the client have reliable transportation, access to clean water, and economic stability to adapt to extreme weather events? Does the client have access to healthy and affordable food, particularly during climate-related disruptions that affect supply chains and local markets?
- **Health History:** Are there pre-existing conditions (e.g., diabetes, hypertension, kidney disease), mental health conditions, pregnancy, and medications that increase susceptibility to heat or dehydration, such as diuretics, beta-blockers, antipsychotic medications, and certain stimulants?

Taking this history should be culturally sensitive, recognizing how social norms, traditional practices, and economic pressures shape health behaviors.

## Key Principles for Climate and Health History-Taking

1. Focus on the Greatest Health Threats: Clinical encounters often face time constraints and competing priorities. Health professionals should prioritize inquiry into the greatest health threats posed to the patient, particularly infectious diseases, extreme heat, food and water insecurity, and poor air quality. Identifying the most immediate and relevant threat ensures efficient and impactful history-taking.
2. Take a Holistic Approach: Using the One Health framework—recognizing the interconnectedness of human, animal, plant, and environmental health—can help clinicians frame questions to better understand the broad effects of climate change on a patient’s health status and vulnerabilities.
3. Consider Cultural Influences: Cultural practices, norms, and perceptions influence environmental exposures and health behaviors. For example, attitudes toward hydration and outdoor labor practices may modify risk for heat-related illnesses. Clinicians should inquire sensitively into cultural contexts to better interpret exposures and risks, including the use of medicinal plants and other traditional care practices.
4. Link Climate-Related Exposures with Health Conditions: Clinicians should actively assess how a patient’s climate exposures are causally or symptomatically linked to both pre-existing and newly diagnosed conditions. For example, extreme heat can exacerbate chronic cardiovascular or kidney disease, while flooding may increase exposure to infectious agents.
5. Inquire about Mental Health Concerns: Climate change impacts—such as extreme weather, food and water insecurity, and forced migration—can trigger psychological distress, anxiety, depression, and trauma-related disorders. Incorporating mental health screening questions into climate histories is essential to fully evaluate a patient’s vulnerability.
6. Assess Changes in Medication Use: Climate events can disrupt medication access (e.g., due to transportation barriers or power outages) and alter medication pharmacokinetics, particularly in cases of dehydration or declining kidney function. Inquire about recent disruptions in medication access, adherence challenges, and any changes in dosage or side effects, especially among patients with cardiovascular disease, diabetes, or chronic kidney disease.

## The Role of Community Health Workers (CHWs)

CHWs, operating across three categories, complement clinical care by extending climate health outreach, education, and patient navigation.

- Category 1 CHWs focus on community-level outreach, health promotion, and education, visiting homes, participating in community events, and promoting awareness of climate-related health risks like heat illness.
- Category 2 CHWs are embedded within primary care clinics and community health organizations, where they facilitate access to clinical services, connect patients, e.g., with cooling centers and hydration resources, and support community partnerships to address other climate-related adaptation needs.
- Category 3 CHWs, preferably with a nurse aid background, receive disease- or condition-specific training, enabling them to support targeted patient populations, such as managing chronic conditions worsened by heat, providing disease-specific education (e.g., managing dengue risk during flood seasons), and coordinating follow-up care.

**Figure 1:**
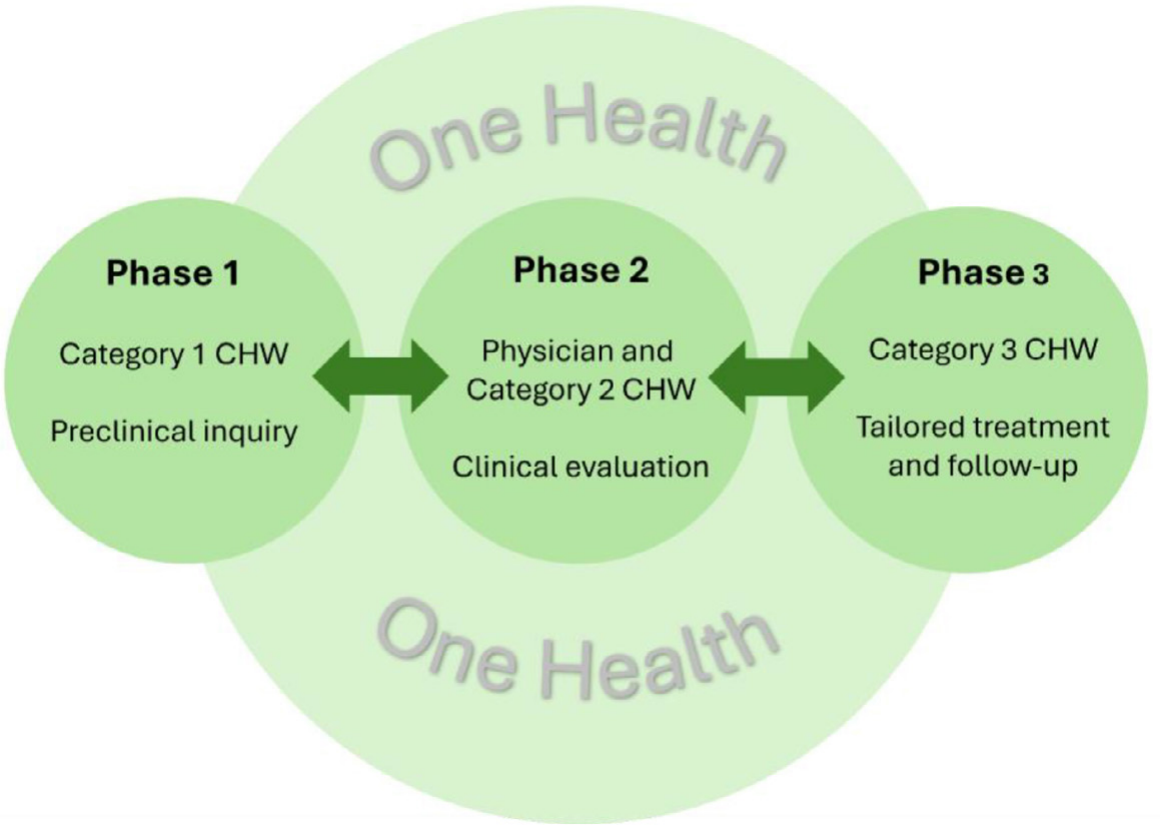
Role of CHWs in climate and health history-taking.

Each CHW category plays a distinct and complementary role in a holistic, community-centered approach to climate and health resilience. Figure 1 illustrates the embedded role of CHWs in climate and health history-taking.

## Physician Responsibilities in Climate and Health History-Taking

Physicians and other primary care providers play a critical role in climate and health history-taking by:

- Incorporating targeted climate exposure questions into initial assessments.
- Identifying patients at elevated risk for climate-sensitive conditions.
- Documenting exposures and vulnerabilities in health records.
- Tailoring health advice and management plans to include climate adaptation strategies.
- Assuring continuity of care and services for high-risk clients by engaging CHWs in all three tiers in clinical and community settings for public health interventions including community education and support.

Climate-informed assessment requires evaluating the patient’s physical environment, access to cooling or heating, availability of safe drinking water, and underlying mobility limitations. Providers should ask about recent exposures to heat, flooding, wildfire smoke, or vector-borne disease environments. It is also important to assess if the patient has an emergency plan, including family communication strategies, access to medications, water storage, backup power, and evacuation arrangements [15].

In LMICs, nurses, midwives, and other health professionals deliver a substantial proportion of primary care. Given the interdisciplinary nature of climate-related health risks, an interprofessional approach involving nurses, CHWs, environmental health officers, and physicians is essential for effective adaptation strategies.

Table 1 provides a list of clinical resources that may inform climate and health history-taking efforts.

**Table 1:** Climate and Health Clinical Resources.

ORGANIZATION	RESOURCE
World Health Organization	Pediatric Environmental History Green Page Questionnaire [16]
	Climate and Health Training Modules for Healthcare Providers [17]
Centers for Disease Control and Prevention	Clinical Guidance for Heat Health [18]
	CHILL’D-OUT Heat and Health Screening Questionnaire [18]
	HeatRisk Tool [18]
Pan American Health Organization	Climate Change for Health Professionals: A Pocket Book [19]
	Climate Change and Health Course for Health Professionals [20]
Global Consortium on Climate and Health Education	Climate and Health Course Repository [21]
Climate Resources for Health Education	Database of Instructional Resources [21]
	Climate and Health Implementation Guide [21]
Climate for Health	Climate Action Guides [22]
	Moving Forward: A Guide for Health Professionals to Build Momentum on Climate Action [22]
	Climate Ambassador Training; Extreme Heat & Health Training [22]
CAFE Research Coordinating Center	Climate Educational Resource Hub [23]
National Collaborating Centre for Environmental Health	Extreme Heat Health Check Tool [24]
Americares	Climate Resilience for Frontline Clinics Toolkit [15]
	Wildfire Smoke and Heat-Health Action Plans [15]
Medicine For a Changing Planet	Environmental Case Studies and Instructional Tools [25]

## Case Study

### Patient profile

Lawrence is a 45-year-old male who discloses symptoms that relate to acute kidney injury (AKI). He complains of the following:

- Decreased urine output
- Swelling in his legs and ankles
- Fatigue and weakness
- Nausea and vomiting
- Confusion and difficulty concentrating

### Phase 1 assessment: preclinical inquiry (Category 1 CHW)

A Category 1 CHW conducts an initial home or community visit to engage Lawrence in a preclinical conversation:

## Living Environment

- *What type of work do you do?*
- *What are some of your hobbies or activities you like to do outside of work?*
- *If the patient has outdoor occupations or hobbies, ask:*
  - *Where do you work?*
  - *What are the day-to-day activities you do as part of your job?*
  - *At what times of the day do you work?*

## Environmental Exposures: Extreme Heat

- *Pre-Screening: How many hours per day are you outside in the sun? At what time are you outside in the sun?*
- *Do you protect yourself against the sun when you are outside?*
  - *If yes, how? (i.e., sunscreen, hats, long pants and sleeves).*
- *Do you ever feel overheated during your daily outside activities?*
- *Do you have air conditioning (AC) at home, at work, in your car?*
- *Are there cooling stations with AC in your neighborhood you can use when you feel hot?*

Through culturally sensitive dialogue, the CHW uncovers that Lawrence works 10 hours a day, 5 days a week in outdoor construction, wears long clothing, and often skips breaks due to community work ethics. The CHW also notes Lawrence’s positive mental health.

### Phase 2 assessment: clinical evaluation (physician and Category 2 CHW)

Lawrence visits the primary care clinic where the physician, supported by a Category 2 CHW embedded in the clinic, explores the clinical implications of his exposure. The physician explains the link between heat exposure and AKI, emphasizing how dehydration reduces kidney blood flow and the risks of chronic kidney disease. The follow-up questions include:

- *Did you have any of the following now, or in the past: sweating, headache, dizziness, sick to your stomach, breathing problems, feeling not able to think clearly? If yes: explore cardiovascular and respiratory disease symptoms*
- *Did you experience sunburn, heat rashes, heat exhaustion, heat cramps or heatstroke in the past?*
- *If any, what medications do you use?*

Lawrence discloses he does often experience sweating, dizziness, and nausea about twice a month. He does not currently take any medication.

The Category 2 CHW assists by:

- Facilitating communication between Lawrence and the medical team
- Helping translate clinical advice into actionable strategies
- Connecting Lawrence to community resources such as cooling and hydration stations

### Tailored treatment and follow-up (Category 3 CHW)

Understanding Lawrence’s economic and social constraints, a Category 3 CHW, trained in chronic disease management, supports him to adhere to clinical directions provided by the physician, as well as to implement practical interventions while respecting his need to maintain his job. The CHW:

- Encourages Lawrence to begin taking breaks in the nearest cooling center for meals
- Advises on hourly hydration strategies to prevent worsening kidney injury
- Provides education on symptoms that require urgent attention
- Monitors adherence to hydration and rest through regular check-ins
- Coordinates follow-up appointments and mental health screening
- Collaborates closely with the medical team to address any emerging clinical needs

## Discussion

Climate change is reshaping patterns of disease, environmental exposures, and vulnerability at a rapid pace, particularly in LMICs. Frontline health workers, including physicians, nurses, and physician assistants, must adapt clinical practice to recognize and address these new risks. Systematic climate and health history-taking offers an actionable, low-cost strategy to build climate resilience into healthcare delivery. However, physicians cannot address this challenge alone. CHWs are crucial partners in extending climate-sensitive care before, during, and beyond the clinic and into communities where adaptation is most urgently needed. CHWs are uniquely positioned to identify at-risk individuals early, provide tailored education, and bridge gaps between healthcare systems and vulnerable populations, e.g., by supporting adherence to treatment and preventive measures.

Integrating CHWs into climate and health history workflows not only improves early detection and prevention but also empowers communities to adapt from within, fostering greater trust, relevance, and sustainability in health interventions.

To effectively implement climate and health history-taking, both physicians and CHWs require targeted training. Clinicians must be equipped to recognize climate-sensitive health conditions, integrate One Health principles into clinical reasoning, and engage in culturally sensitive communication about climate risks. Similarly, CHWs should be trained to conduct climate-focused screenings, identify early warning signs of climate-related illness, and support community-level adaptation strategies. Building this workforce capacity is critical to embedding climate resilience into healthcare systems, particularly in low-resource settings.
